# Impact of stigma and stigma-focused interventions on screening and treatment outcomes in cancer patients

**DOI:** 10.3332/ecancer.2021.1308

**Published:** 2021-10-25

**Authors:** Elizabeth O Akin-Odanye, Anisah J Husman

**Affiliations:** 1 Department of Clinical Psychology, University College Hospital, Queen Elizabeth Road, Oritamefa, Ibadan, Oyo State, 200212, Nigeria; 2 College of Health Professions and Sciences, University of Central Florida, 4000 Central Florida Blvd, Orlando, FL 32816, USA

**Keywords:** cancer, stigma, systematic review

## Abstract

**Background:**

Stigma is known to negatively influence cancer patients’ psychosocial behaviour and treatment outcomes. The aim of this study was to systematically review the current data on cancer-related stigma across different populations and identify effective interventions used to address it.

**Methodology:**

The protocol, search, appraisal, synthesis, analysis and reporting framework was used for conducting this systematic literature review. CINAHL, PubMed, PsycINFO and Google Scholar databases were searched using the different combination of keywords that include ‘cancer stigma’. Articles publication period was set for 2010–2020. A total of 54 articles (31 quantitative, 19 qualitative, 2 mixed methods and 2 scoping reviews) that met inclusion criteria were reviewed out of the 958 articles initially identified. Quality assessment of included studies revealed the studies had varying levels of methodological quality. Extracted data were organised and narratively analysed.

**Results:**

Cancer stigma was expressed across different segments of the society including amongst the elites and healthcare providers. Developing countries had higher rates of stigma reported and experience of stigma varied by cancer type. Cancer was consistently associated with imminent death in all studies reviewed. Cancer patients experiencing stigma were more inclined to conceal their diagnosis and to seek medical help later. Whilst cancer stigma majorly resulted in negative psychosocial outcomes in patients, there were also instances of posttraumatic growth emanating from the stigma experienced. Literature on cancer-related stigma interventions was scant.

**Conclusion:**

Cancer related stigma remains high in both clinical settings and amongst the general public. There is need for more interventions to combat cancer stigma and its effect in both patient and non-patient population. Anti-cancer public enlightenment campaigns should be sensitively designed to not further fuel stigma against patients with certain types of cancers.

## Background

Stigma is a discrediting characteristic that reduces a person ‘from a whole and usual person to a tainted, discounted one’ [25]. ‘It is a process whereby the societal reaction to a person or his condition negatively alters the individual’s normal identity’ and devalues him in the eyes of others [39, 53]. Health-related stigma refers to stigmatisation of an illness, which can be applied to an individual or a group of people with the illness, as well as to the illness more generally [61]. Illness stigmatisation is not stable, but is influenced by social attitudes that differ across cultures and change over time [16]. Most of the literature exploring health-related stigma has focused on a small group of illnesses: cancer, disabilities, leprosy, epilepsy, HIV/AIDS, tuberculosis and mental illness [1, 14, 76]. 

Cancer-related stigma has especially drawn attention to the detrimental burden of social views on the lives of patients [4, 7, 17, 83]. Patients sometimes feel avoided by others once they have received a cancer diagnosis [33] and fear of stigmatisation can be a barrier to disclosure of a cancer diagnosis [70]. Cancer stigma studies and reviews have tended to focus on single cancer types such as lung cancer [9] or specific aspects of cancer patients lives that could be affected by cancer such as work. Few studies have explored cancer stigma across multiple settings although such studies could inform a better understanding of how cancer stigma differ across different populations well as aid in the development of more targeted interventions to combat cancer stigma and its effect on health outcomes.

So far, it is not very clear how stigma is experienced by patients with different types of cancer, how it affects screening and health seeking behaviour and outcomes as well as available interventions to help cancer patients cope with perceived and internalised cancer. Hence, this systematic review was conducted to help inform the current state of stigma, its impact and interventions across multiple cancer types and geographical spread.

## Methodology

Protocol: This systematic literature review (SLR) was done using the protocol, search, appraisal, synthesis, analysis and reporting framework [89]. The review scope was defined using the framework of Population, Intervention, Comparison, Outcome and Context (PICOC). The SLR sought to address the following research questions:

1. How is cancer stigma expressed and experienced?
2. How does cancer stigma affect cancer screening and health seeking behaviour?
3. What is the impact of stigma on cancer patients’ psychosocial and health outcomes?
4. How efficacious are available cancer stigma-focused interventions?

### Search

The keywords ‘cancer stigma’, ‘cancer stigma interventions’, ‘cancer stigma impact’, ‘cancer stigma effect’, ‘stigma and cancer screening’, ‘Cancer stigma and health seeking behavior’ were used in searching for relevant articles written in English language and restricted to the past 10 years from PubMed, PsycINFO, CINAHL and Google Scholar. The relevant studies were identified based on pre-specified inclusion and exclusion criteria.

#### Inclusion criteria

Studies with the under listed features will be included in the systematic review

1. Studies describing the experience or expression of cancer stigma in both patient and non-patient population.
2. Studies reporting effect of stigma on cancer screening and health seeking behaviour.
3. Studies reporting impact of cancer stigma on psychosocial, behavioural and treatment outcomes in cancer patients.
4. Studies reporting cancer stigma-focused interventions and their effectiveness.
5. Studies must have been published between 2010 and 2020 and must be written in English.
6. The title of study must include the words ‘cancer’ and ‘stigma’.

#### Exclusion criteria

Studies with the under listed features will be excluded from the systematic review

1. Studies not reflecting both ‘cancer’ and ‘stigma’.
2. Studies reflecting both ‘cancer’ and ‘stigma’ in their titles but whose contents reveal that were not primarily focused on cancer stigma.
3. Studies reflecting the words ‘cancer’ and ‘stigma’ in their titles that were only focused on validation of instruments used for assessing stigma.

### Appraisal

**Figure 1.**
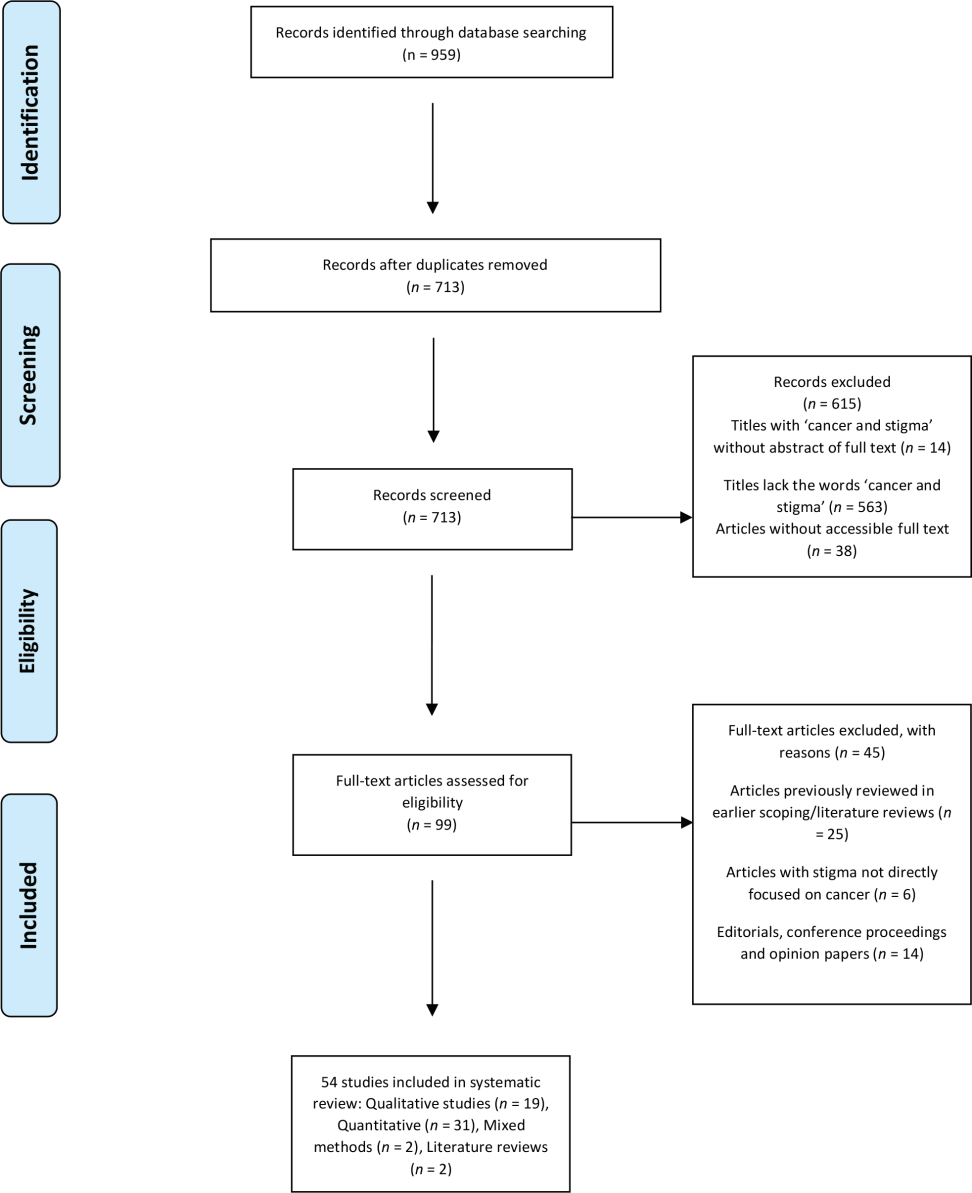
The Preferred Reporting Items for Systematic reviews and Meta-Analyses flowchart of studies included in the review.

In this phase, all selected papers were screened for relevance based on the objectives set out for this review work. All papers that met the earlier stated inclusion criteria were selected for further content assessments. Papers that were opinion papers without clear methodology and extended abstracts were removed. The flow diagram on Figure 1 shows the screening process for papers selected. Of the 959 peer reviewed studies originally gathered, 54 (5.63%) met the inclusion criteria and were included in this review.

Quality assessment of the selected studies was conducted using the mixed methods appraisal tool (MMAT) version 2018 [36] for all non-systematic review studies. Each scoping literature review in this study was assessed using five quality assessment (QA) questions (see Table 1). All assessment questions were responded to using the options: Yes, Can’t tell or No. Each of the included qualitative study had a ‘Yes’ response to all the assessment questions, indicating they are of high quality. Amongst the quantitative studies, only 1 study had a ‘Yes’ response to all the assessment questions, whilst 16 studies had between three and four ‘Yes’ responses to all the assessment questions. Out of the two mixed methods study, one had all ‘Yes’ responses whilst the other had no ‘Yes’ response because only the quantitative aspect of the study was reported. The scoping reviews had four ‘Yes’ responses each. All studies were included in the review irrespective of adjudged quality (Details in Supplementary Data, Table S1).

### Synthesis

To address the objectives of conducting the SLR, the relevant information from the articles such as names of authors, years of publication, country, study population, method of analysis (quantitative, qualitative, mixed or systematic review), aims, stigma scales and results were extracted into an excel spreadsheet for data processing. The extracted information is presented in Table 2.

**Figure 2.**
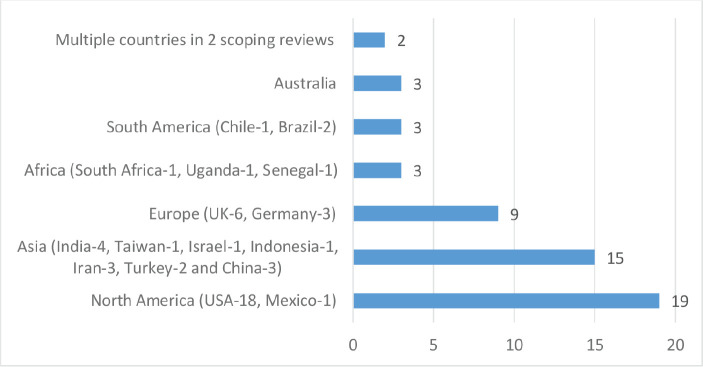
Number of included studies by continent.

**Figure 3.**
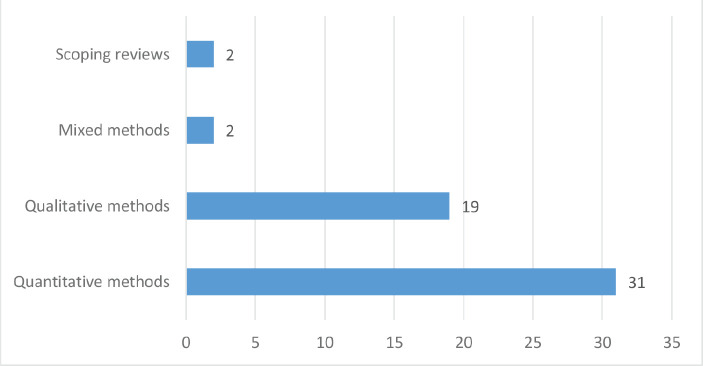
Methods used by included studies.

**Figure 4.**
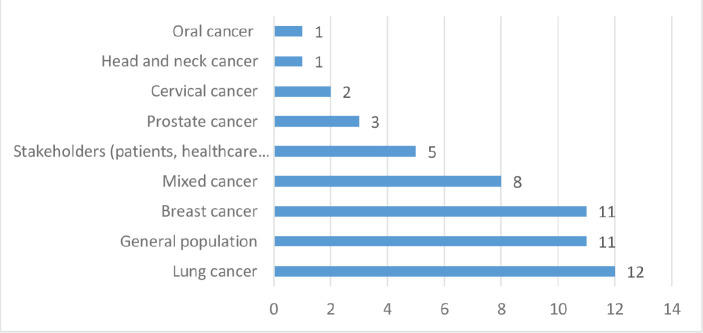
Number of included studies by studied population or cancer types.

Out of the 54 studies reviewed, 19 were conducted in North America (USA – 18 and Mexico – 1), 3 in Africa (South Africa – 1, Uganda – 1 and Senegal – 1), 15 in Asia (India – 4, Taiwan – 1, Israel – 1, Indonesia – 1, Iran – 3, Turkey – 2 and China – 3), 9 in Europe (UK – 6 and Germany – 3), 3 in South America (Chile – 1 and Brazil – 2), 3 in Australia and 2 scoping reviews with multiple countries (see Figure 2). Furthermore, 31 (57.41%) studies used quantitative methods of data analysis, 19 (35.19%) were qualitative, 2 (3.70%) mixed methods studies and 2 (3.70%) scoping reviews (see Figure 3). Lung cancer stigma (LCS) in patients was the focus of 12 of the included studies, 11 of the studies were on stigma in breast cancer patients, 11 on cancer stigma perceptions of the general public, 8 on cancer stigma in a mixed cancer population, 5 on cancer stigma perceptions of stakeholders (cancer patients, families and healthcare providers), stigma in prostate cancer patients was the focus of 3 studies, 2 studies were on cervical cancer stigma whilst 1 each was on oral and head and neck cancer (see Figure 4).

The scales used for assessing stigma in the included studies were Cancer Stigma Scale (CASS) by Marlow and Wardle [45], Social Impact Scale (SIS) by Fife and Wright [24], Body Image After Breast Cancer Questionnaire (BIABCQ) by Baxter et al [5], Self-Stigma Scale-Short Form (SSS) adapted for breast cancer survivors (BCS) by Mak and Cheung [43], Lung Cancer Stigma Scale (LuCaSS) by Maggio [43], the Lung Cancer Stigma Inventory (LCSI) by Hamann *et al* [31], Cataldo Lung Cancer Stigma Scale (CLCSS) by Cataldo *et al* [10], six-item stigma scale by Phelan *et al* [59], Turkish version of the ‘questionnaire for measuring attitudes toward cancer (cancer stigma) – patient version’, by Cho *et al* [13], discrimination and stigma scale (Adapted from Thornicroft *et al* [68]), German version of the Social Impact Scale (SIS-D) by Eichhorn *et al* [20], Cancer Stigma Index (CSI) by Edelen *et al* [19] and some studies used items adapted from different scales.

### Analysis

#### Expression and experience of cancer stigma

**• Ubiquitous nature of cancer stigma**

The prevalence of high cancer stigma in the studies reviewed range from 26.1% amongst a sample of patients with different types of cancer in Iran Shiri *et al* [64] to 35.5% amongst lung cancer patients in the USA [37]. The included articles showed that the stigma attached to cancer and cancer patients is expressed and experienced from different segments of the society, such as the general public [6, 54, 56, 58, 62, 63, 72], elites [50], media and advertising agencies [18, 27, 55, 80], healthcare providers [28, 34]), policy makers [49] and friends and family members [63, 101].

Cancer stigma and anticipated discrimination often began from being labelled based on physical appearance of perceived signs of cancer [58]. Furthermore, the cost of cancer treatment paired with poor prognosis led to a stigma of draining family resources and being shamed by in-laws for bringing cancer into the family [29, 46]. Stigma induced acts of social and physical isolation include ceasing to invite persons with cancer to social events, physical separation of their personal effects or sleeping quarters, neglect by spouse or family and friends, verbal stigma ranging from gossip to outright abuse were copiously described in some studies [92, 54, 72]. However, narratives of family support also emerged alongside the presence of prevalent and harmful stigma in some studies [29, 46, 54, 92].

• **Stigma and type of cancer**

Women with breast cancer described the burden of being expected to be positive despite the continued association of cancer with death and how they sometimes had to downplay their private suffering to present a positive front to others [72]. Amongst these women, being less educated and opting for breast conservation surgery [71], being on chemotherapy, having depressive symptoms with high levels of ambivalence over emotional expression and intrusive thoughts [74] were significantly associated with higher self-stigma. Furthermore, there was significant inverse relationship between annual household income [74] and time since diagnosis 100] with self-stigma in patients with breast cancer. Patients with breast and prostate cancers appeared less stigmatised [18, 22, 55, 86, 87]. Respondents in some of the studies opined that the public regards breast cancer as a ‘blameless’ disease [18] that people get ‘pink, warm, and fuzzy’ about [55]. Nevertheless, like with breast cancer patients, time since diagnosis was found to be inversely correlated with prostate cancer patients’ stigma score in a prospective study [88].

The experience of cancer stigma related to shame and blame appeared highest amongst patients with lung and cervical cancers due to their links with smoking [55, 81] and STIs [18, 62], respectively. Studies reported significantly higher levels of stigma in lung cancer patients with younger age, depression, greater social deprivation/constraints, unemployment, higher negative changes following diagnosis, higher cancer stage, perceived blame from others, concealment of cancer diagnosis, lower coping self-efficacy, poor self-esteem and being an ever smoker [93, 40, 43, 57, 81, 84]. However, findings on the relationship between smoking status and level of stigma are inconsistent as some studies did not find significant association between smoking status and lung cancer stigma [8, 40].

Women with cervical cancer, on the other hand, could not risk others ﬁnding out they had cancer, for fear of being considered ‘spoiled’ or ‘ruined’ [28] and some felt so ashamed and embarrassed that they tell others they had uterine cancer instead of cervical cancer to avoid being the centre of gossip [18]. A similar experience was shared by men with breast cancer who would rather use the term ‘çhest cancer’ in describing their diagnosis [79].

In patients with mixed cancer types, negative attitudes toward cancer were associated with being older than 60-year-old, possessing higher education, earning low income and feeling socially excluded [90]. Some inconsistencies were, however, found in the association between educational level and stigma in the included studies amongst patients with mixed cancer types [64, 90].

• **Impact of stigma on physician–patients–family cancer communications**

Stigma associated with communicating a cancer diagnosis is enacted by the clinicians who choose to use euphemisms like ‘inﬂammation’ or ‘wound’ rather than the word ‘cancer’ [28]. The doctors in conspiracy with family and friends also, often failed to fully disclose to the patient their diagnosis which sometimes resulted in patients’s feeling worried about their condition or becoming so hopeful for a cure that they invest on expensive treatment that further plunge them into debt [92, 34, 54].

• **Impact of cancer related stigma on family members of cancer patients**

Apart from cancer patients, members of their families and caregivers also experience cancer-related stigma. Caregivers of lung cancer patients, for instance, were found to experience stigma by association as members of the patients support system and reported feeling invisible and unsupported [55]. Also, in Uganda, children of women with cancer of marriageable age risk not getting someone to marry due to the rollover cancer stigma to the children [46].

• **Stigma perceived by cancer survivors**

Cancer survivors also experienced the structural dimension of stigma in terms of the lack of policies to protect those affected by cancer and the dearth of resources for research, information and support services for patients with some types of cancers [18, 49]. A good number of MBC reported experiencing sexual stigmatisation in the process of accessing cancer care because nothing within the clinical setting points to the possibility of men fitting in, thereby making them feel unwelcomed [48, 79].

• **Coping with cancer stigma**

Women with breast cancer often cope with public stigma and self‐stigmatisation through social support, prayers, maintaining positive outlook, acceptance of diagnosis and ignoring negative comments [46, 92]. The women with cervical cancer in Recife, Brazil accepted and coped with their lot in life by drawing on stigmatising metaphors to construct narratives that would help them understand the relationship between their new, ill, and, therefore, different selves and the world they have always known [28]. However, most patients and their families in a stakeholders’ study were reported to cope via non-disclosure of cancer and limiting contact with others in other to avoid being an object of sympathy from others and to avoid getting misleading information that may discourage them [50].

• **Possible explanations for the experience and expression of cancer stigma**

Some authors attempted to make sense of the reasons behind stigma against cancer and those affected by cancer. Amongst the reasons adduced were: blame apportioning, disgust propensity, gender differences, having not experienced cancer or the behaviours that are thought to cause cancer and being a member of an ethnic minority [3, 6, 41, 56, 62, 77]. The role of gender in cancer stigma is not consistent in literature. Whilst women were reported to be more likely than men to express stigmatic behaviours against people with cancer in a study [56], men were more probable culprits in others [62, 77].

### Perceived impact of cancer stigma on screening and patients’ health seeking behaviour

The notion of cancer as a terrible disease that is linked with death, dread, doubt, distress, shame and blame [33, 35, 50, 64, 65, 77, 78, 92, 92] lead to disease concealment to avoid being judged, delayed treatment and use of traditional healers rather than biomedical treatment [18, 29, 40, 58, 81, 63].

In the study from Uganda, BCS described breast cancer‐related stigma as a barrier to care even before a diagnosis is made as the stigma could affect their marriage and family [46]. Similarly, in an Indian study, breast and cervical cancer patients stated that cancer stigma was present in their lives and communities, was a feared outcome of a cancer diagnosis and a barrier to cervical screening, early diagnosis and treatment seeking for women even as symptoms worsened [54]. Also in the UK, higher total cancer stigma was significantly associated with less likelihood of screening for cervical, breast and colorectal cancers [77]. In the USA, a statistically significant positive correlation was found between perceived lung cancer stigma and delayed medical help seeking [8]. It thus seems that irrespective of cancer type, across different geographical and cultural divides, cancer stigma presents as a ubiquitous barrier to health seeking behaviour. However, an Australian study reported no significant association between lung cancer stigma and help-seeking behaviours in terms of accessing available hospital services to support their care [60]. Beyond stigma, respondents in a study in India hold the view that the cost of treatment, reliance on alternative medicine, fear of being diagnosed and fatalistic beliefs were the main barriers to health seeking behaviour [29]. However, these reasons, especially fear of being diagnosed and fatalistic beliefs appear to have some indirect link with internalised stigma.

### Influence of cancer related stigma on psychosocial, behavioural and health outcomes

The social impact of cancer stigma is strongly enacted in the workplace context affecting cancer survivors’ employability due to high likelihood of hiring discrimination, job loss and limited career advancement [66]. Whilst ability to return to work even if in a limited capacity can help mend the disruption caused by the illness, inability to resume work with the inevitable reliance on others and welfare benefits could confer a stigmatised identity of helplessness in individuals who prior to their diagnosis were conscientious workers and providers for other [49].

Psychologically, a USA based online survey that assessed public perceptions and stigma due to listing cancer as the cause of death in celebrity obituary, found that lung cancer as a cause of death (as compared with liver cancer or death by an undisclosed cause) increased both anxiety and sadness, with anxiety then leading to increased origin-related stigma [51]. The results of this study imply that stigmatization leads to increased symptoms of grief when it is transferred into negative discriminatory behavior, which is also referred to as ‘enacted stigma’. Social stigma attached to cancer, which manifests itself in negative discrimination, may keep patients from disclosing their illness and cause them to ‘suffer in silence’ by isolating them from social interactions and interrupting the resolution of grief. Thus enacted stigma increases the likelihood of negative illness perception which if central to one’s self-definition may result in increased the grief symptomatology. [26]. Furthermore, it was found that within person, fluctuations in stigma were related to social and role functioning in patients with lung cancer, regardless of physical symptoms and were not carried over to the next day [65]. Evidence of positive correlation between stigma and anxiety [11, 88], depression [11, 23, 90, 93], higher self-perceived burden [88], symptom burden [93, 43] and affective interferences [37] were also reported. Body image and social support were found to mediate the relationship between stigma and depression in cancer patients [23, 43].

Studies showed that stigma had negative relationship with QoL and/or some of its subscales in patients with oral cancer, prostate cancer, breast cancer, lung cancer and in a group of patients with different cancer type [22, 37, 52, 84–87, 89, 91]. It also had an inverse relationship with hope and social support [91], posttraumatic growth [85], good healthcare provider communication [81], NK subset of cellular immunity, doctors’ empathy and patients’ self-efficacy [88]. Pain intensity was found to mediate the relationship between stigma and QoL in breast cancer patients [52].

Unexpectedly, some studies reported findings of posttraumatic growth borne out of patients accepting their experience of cancer and the stigma that comes with it [18, 28, 69]. In the USA, one of the positive consequences of cervical cancer-related stigma that some women witnessed and experienced became a powerful catalyst for their involvement in the awareness-raising movement and advocacy to enlighten people about the devastation of cervical cancer and the true prevalence of human papilloma virus (HPV) [18]. The women with cervical cancer in Brazil accepted their lot through reframing and the use of metaphors because for many of them the story of sexual impurity and cervical cancer was not just about stigma and shame but also about the possibility of hope for redemption and a journey back in time to health and sexual purity which their vaginal narrowing and dryness caused by radiation therapy would offer, suggesting that they would not just be healed, but they would become ‘virgins’ again [28]. Also, acceptance triggered an awakening of new life interpretations and psychological growth in head and neck cancer patients in Australia who through their personal experience of stigma were able to acquire previously unfelt empathetic understanding and altruism towards others with cancer [69].

### Interventions addressing cancer stigma and their effectiveness

There was a dearth of studies on interventions addressing cancer stigma in the reviewed articles. Only two cancer-related stigma intervention studies were found. The first was an online intervention in the UK conducted to increase cervical cancer screening intention and by extension screening uptake [80]. The authors used public stigma toward people who had not been screened for cervical cancer as a tool to craft emotive narrative within news articles for respondents to read before responding to items on the study instruments. It was found that the emotive narrative within news articles was more effective in enhancing the willingness of women to attend a cervical screening appointment compared to factual information or no information [80]. The authors opined that emotive narratives within news articles can promote screening through increases public stigma.

The other study on cancer-related stigma intervention was located amongst studies reviewed by Webb *et al* [81]. The authors of the study used telephone acceptance-focused cognitive behavioural intervention to address stigma in people with lung cancer. The pre- and post-test outcome measures were of psychological and cancer-specific distress, depression, health-related stigma and QoL. At post-intervention, significant decrease was reported for psychological and cancer-specific distress, depression and health-related stigma but there was also a decline in QoL [94]. 

### Implications of the present review

**Implications for clinicians**: The negative impact of cancer stigma on the clinician–patient–family communication reported in this review underscores the need for psychosocial interventions with potential to eliminate barriers to communication and improve patients and clinicians’ communication skills within oncology settings. Furthermore, clinicians ought to recognise that the cancer stigma is a multifaceted phenomenon that varies by type of cancer across factors such as actual or perceived causes, treatments and outcomes as well as the sociocultural environment within which the disease is been experienced. Such realisation would help the attending clinician sensitively tailor consultation to prevent and mitigate both individual and structural level stigma.

**Implications for future researchers:** There is dearth of interventions for reducing or managing cancer-related stigma both in clinical settings and in the community. Thus, understanding patients and caregiver strategies for coping with cancer stigma as well as what works in preventing or managing it requires further research. It is also noteworthy to observe the paucity of research on social impact of cancer stigma from low and middle income countries especially of Africa where the global cancer burden is huge. This demands the attention of researchers from these regions as well as that of research funding agencies. Further still, more focus could be directed at the stigmatising aspects of cancer and its treatment in future research and in the design of evidence-based psychosocial interventions.

**Implications for service development and policy:** The findings of the role of the media in innocently promoting stigma against certain cancers as well as the perceived discrimination in the workplace due to cancer diagnosis and structural level stigma in how social and healthcare services are provided have implication for service development and policy. Whilst some countries such as the USA [2] may have legislations to protect cancer patients from work place discrimination, there is need for policy makers in settings where this does not exist to enact policies that further protect the interest of those living with cancer in terms of job hiring, placement and career progression. There is also need for policies to regulate public health communication pro cesses to avert the unintentional labelling and stigmatising of patients [30]. Also, service providers should create destigmatising services (such as support groups and information services) for cancer patients irrespective of gender and cancer types.

## Discussion

The experience of cancer stigma was pervasive in most of the studies included in our review. This seemed to agree with the notion of cancer as one of the most stigmatised disease conditions in many societies [19]. The fear of cancer stigma and associated fatalism, shame and anxiety prevent many from engaging in cancer prevention practices, screening and seeking health services [19, 32, 67]. Patients with breast and prostate cancers were less stigmatised compared to those with lung and cervical cancer mainly due to the perception that the latter are self-caused through wrong choices [21, 44] and as such are often blamed for their condition [12].

The use of both adaptive coping strategies such as cognitive reframing, acceptance, religious faith and ignoring negative comments [28, 46, 92] and dysfunctional coping techniques like non-disclosure of diagnosis and avoidance [50] were used by cancer patients to deal with stigma. However, utilising maladaptive coping strategies have been significantly associated with depressive symptoms [59, 95]. 

Furthermore, cancer stigma negatively impact communication between patients, physician and family members. It also resulted in poorer psychosocial and health outcome for cancer patients as well as work place discrimination, poor QoL, increased depression and anxiety. These findings which were also reported in earlier studies [94, 15, 24, 35] are begging for the development of more evidence-based psychosocial and clinical interventions to reduce stigma. Psychosocial interventions may focus on altering targeted health problems and behaviours using behaviour change interventions and social policies that refute the tendency of perpetrators to stigmatise, and enhance resilience in the stigmatised thereby making them less vulnerable to the negative impact of stigma [35, 82]. Clinical interventions may emphasise the use of biomedical procedures to help reduce the consequences of cancer treatments, thereby reducing stigma associated with certain cancers. For instance, the course of cervical cancer disease and the treatments for it like most gynaecological cancers can compromise fertility and reproductive capacity in affected women further fuelling stigma in this group. However, offering fertility-sparing techniques to young unmarried women with gynaecological cancers may contribute to not only improving the QoL of these women [38, 75] but also to reducing the stigma associated with their diagnosis.

## Conclusion

Stigma contributes to the burden of illness for cancer patients and their family members. The findings of this study have provided invaluable direction for developing interventions to tackle cancer stigma in the general populace, amongst clinicians providing care for cancer patients and amongst the cancer patients themselves. The general public need to be better educated about cancer as a non-communicable disease and the place of appropriate screening for early detection to enhance better treatment outcome. Public enlightenment campaigns to promote the adoption of modifiable cancer risk preventive behaviours should be designed to not further escalate cancer stigma. Healthcare providers should be involved in ongoing communication training to enable them act and speak in ways that are destigmatised when relating with those affected by cancer. To prevent or overcome self-stigmatisation, interventions to help cancer patients accept their new identity and improve their body image should be developed [23]. Furthermore, legal instruments as well as administrative and clinical structures should be created to protect the interests of cancer patients in the workplace, at the health facilities where they receive care or at the government or non-governmental agencies where they seek social support for their care.

## Strengths and limitations

Our study has some limitations. Firstly, our inclusion criteria that only articles with the terms ‘cancer’ and ‘stigma’ would be reviewed may increase the likelihood of omitting studies without these two key words occurring together in the title but that may have contents that are relevant to cancer stigma. Also, although we endeavoured to include all relevant studies with the words ‘cancer’ and ‘stigma’, there were several studies whose full text contents could not be accessed and we cannot say with certainty that all full text articles available in the public domain were accessed and assessed. However, we did not limit the types of studies reviewed to a specific methodology or a specific population which ensured that a wide variety of studies were reviewed so long as the study was related to cancer stigma.

## Funding declaration

This study was funded by the ReTOOL Program (National Cancer Institute/National Institutes of Health R25CA214225).

## Conflicts of interest statement

The authors have declared that they have no conflicts of interest.

## Figures and Tables

**Table S1.** Methodological QA of all included studies.

**QA of included qualitative studies (*n* = 18 ) using MMAT five questions checklist**						
**S/N**	**Studies**	**Is the qualitative approach appropriate to answer the research question?**	**Are the qualitative data collection methods adequate to address the research question?**	**Are the findings adequately derived from the data?**	**Is the interpretation of results sufficiently substantiated by data?**	**Is there coherence between qualitative data sources, collection, analysis and interpretation?**
1.	Shiri *et al* [63]	Yes	Yes	Yes	Yes	Yes
2.	Threader and McCormack [69]	Yes	Yes	Yes	Yes	Yes
3.	Harding *et al* [34]	Yes	Yes	Yes	Yes	Yes
4.	Moffatt and Noble [49]	Yes	Yes	Yes	Yes	Yes
5.	Tang *et al* [67]	Yes	Yes	Yes	Yes	Yes
6.	Walker and Berry [79]	Yes	Yes	Yes	Yes	Yes
7.	Meacham *et al* [46]	Yes	Yes	Yes	Yes	Yes
8.	Trusson and Pilnick [72]	Yes	Yes	Yes	Yes	Yes
9.	Oystacher *et al* [58]	Yes	Yes	Yes	Yes	Yes
10.	Dyer [18]	Yes	Yes	Yes	Yes	Yes
11.	Gregg [28]	Yes	Yes	Yes	Yes	Yes
12.	Nyblade *et al* [54]	Yes	Yes	Yes	Yes	Yes
13.	Occhipinti *et al* [55]	Yes	Yes	Yes	Yes	Yes
14.	González and Diaz-Castrillón [27]	Yes	Yes	Yes	Yes	Yes
15.	Machado *et al* [42]	Yes	Yes	Yes	Yes	Yes
16.	Luberto *et al* [41]	Yes	Yes	Yes	Yes	Yes
17.	Gupta *et al* [29]	Yes	Yes	Yes	Yes	Yes
18.	Solikhah *et al* [92]	Yes	Yes	Yes	Yes	Yes
19.	Mohabbat-bahar *et al* [50]	Yes	Yes	Yes	Yes	Yes
**Quality assessment of included mixed methods studies (*n* = 2) using MMAT five questions checklist**						
**S/N**	**Studies**	**Is there an adequate rationale for using a mixed methods design to address the research question?**	**Are the different components of the study effectively integrated to answer the research question?**	**Are the outputs of the integration of qualitative and quantitative components adequately interpreted?**	**Are divergences and inconsistencies between quantitative and qualitative results adequately addressed?**	**Do the different components of the study adhere to the quality criteria of each tradition of the methods involved?**
1.	Midding *et al* [47]	Yes	Yes	Yes	Yes	Yes
2.	Carter-Harris [8]	No	Can’t tell^a^	Can’t tell^a^	Can’t tell^a^	Can’t tell^a^
**Quality assessment of included quantitative descriptive studies (*n* = 28) using MMAT five questions checklist**						
**S/N**	**Studies**	**Is the sampling strategy relevant to address the research question?**	**Is the sample representative of the target population?**	**Are the measurements appropriate?**	**Is the risk of nonresponse bias low?**	**Is the statistical analysis appropriate to answer the research question?**
1.	Vrinten *et al* [77]	Yes	Yes	Yes	Yes	Yes
2.	Azlan *et al* [3] (Phase 1)	Yes	Can’t tell	Yes	Can’t tell	Yes
3.	Zhang *et al* [91]	Yes	Can’t tell	Yes	Yes	Yes
4.	Yang *et al* [88]	Yes	Can’t tell	Yes	Can’t tell	Yes
5.	Wood [86]	No	No	Yes	No	Yes
6.	Wood *et al* [87]	No	No	Yes	No	Yes
7.	Tripathi *et al* [71]	Yes	Can’t tell	Yes	Can’t tell	Yes
8.	Tsai *et al* [74]	No	Can’t tell	Yes	Can’t tell	Yes
9.	Tsai and Lu [73]	No	Can’t tell	Yes	Can’t tell	Yes
10.	Wong *et al* [85]	Yes	Yes	Yes	Can’t tell	Yes
11.	Yeung *et al* [89]	No	No	Yes	Can’t tell	Yes
12.	Maggio [43]	No	No	Yes	Yes	Yes
13.	Cataldo and Brodsky [11]	No	No	Yes	Can’t tell	Yes
14.	Ostroff *et al* [57]	Yes	No	Yes	Yes	Yes
15.	Liu *et al* [40]	No	No	Yes	Yes	Yes
16.	Johnson *et al* [37]	No	No	Yes	No	Yes
17.	Rose *et al* [60]	Yes	No	Yes	Can’t tell	Yes
18.	Williamson *et al* [84]	Yes	No	No	Yes	Yes
19.	Steffen et al [65]	Can’t tell	No	Yes	Yes	Yes
20.	Esser *et al* [23]	Yes	No	Yes	No	Yes
21.	Yilmaz *et al* [90]	No	No	Yes	Can’t tell	Yes
22.	Gökler-Danışman *et al* [26]	Can’t tell	Can’t tell	Yes	Can’t tell	Yes
23.	Ernst *et al* [22]	Yes	Yes	Yes	No	Yes
24.	Shiri *et al* [64]	No	Yes	Yes	Can’t tell	Yes
25.	Ongtengco *et al* [56]	No	No	Yes	Can’t tell	Yes
26.	Myrick [50]	No	No	Yes	Can’t tell	Yes
27.	Bresnahan *et al* [6]	Yes	No	Yes	Can’t tell	Yes
28.	Maguire *et al* [93]	Yes	No	Yes	No	Yes
29.	Nakash *et al* [52]	Can’t tell	Can’t tell	Yes	Can’t tell	Yes
**Quality assessment of included quantitative RCT descriptive studies (*n* = 3) using MMAT**						
**S/N**	**Studies**	**Is randomisation appropriately performed?**	**Are the groups comparable at baseline?**	**Are there complete outcome data?**	**Are outcome assessors blinded to the intervention provided?**	**Did the participants adhere to the assigned intervention?**
1.	Azlan *et al* [3] (Phase 2)	Yes	Yes	Yes	No	Yes
2.	Wearn and Shepherd [80]	Yes	Can’t tell	No	No	Can’t tell
3.	Shepherd and Gerend [62]	Yes	Can’t tell	No	Can’t tell	Yes
**QA of included scoping reviews (*n* = 2) using five questions assessment (QA)**								
**S/N**	**Studies**	**Where the objectives of the review clearly stated?**	**Are the review’s inclusion and exclusion criteria described and appropriate?**	**Is the literature search likely to have covered all relevant studies on the topic?**	**Did the authors assess the quality of included studies?**	**Are the results of the review clear?**
1.	Webb *et al* [81]	Yes	Yes	Yes	No	Yes
2.	Stergiou-Kita *et al* [66]	Yes	Yes	Yes	No	Yes

a Only the quantitative aspect of the study was reported

**Table 1:** Scope of SLR using the PICOC framework to the determined objectives.

Concept	Definition	SLR application
Population	Research papers on cancer-related stigma	Empirical research published in peer review journals on cancer-related stigma in patient and non-patient population. The studies would focus on cancer stigma experience, effect and interventions.
Intervention	Evidence-based strategies for addressing cancer-related stigma	Identifying the existing cancer-related stigma interventions and the gaps that need further research work, such as cancer types for which stigma interventions are not yet available, components of interventions that are yet to be explored as well as modes of delivery.
Comparison	Methods to compare the findings of each cancer-related stigma studies with each other	Differences in the findings of studies based on design, cancer types, country and clinical or non-clinical settings.
Outcome	Strategies to assess the results and gaps identified in the reviewed cancer-related stigma studies	Existing knowledge on specific types of cancer-related stigma, data types, aims and the scale of the studies. Also, studies’ limitations and methodological quality.
Context	Specific settings or population of interest	The geographical distribution of included studies as well the distribution of studies based on respondents’ cancer types or general non-clinical population.

**Table 2.** Characteristics of included studies.

S/N	Author(s), year and country	Study population/design	Aims/stigma scale	Results/stigma level
1.	González and Diaz-Castrillón [27] Chile	General population/qualitative	Performed a discourse analysis of the Chilean Campaign in force during 2014–2016. Focusing on what the campaign promotes in relation to lung cancer, cancer treatments and the causality between smoking and lung cancer. /Not Applicable (NA)	The authors’ analysis led them to conclude that the Chilean Campaign in force during 2014–2016 conceptualised lung cancer as a self-inﬂicted, fatal disease and depicted tobacco use as a synonym to lung cancer, and lung cancer as a synonym of a terminal and mortal condition. It explicitly showed death as a slow, inevitable process, where it is unclear if what kills is tobacco, cancer or medical treatments. They believe that these elements strengthen lung cancer stigma and cancer in general.
2.	Luberto *et al* [41] USA/Online	General population/qualitative	Analysed publicly available social media data to develop a conceptual model explaining individuals’ stigmatic or sympathetic reactions to cancer patients who smoke./NA	The developed conceptual framework suggests that personal experiences with cancer, smoking and statistical literacy influenced beliefs about smoking and cancer, which in turn influenced stigmatic or sympathetic attitudes toward cancer patients who smoke. Individuals with personal smoking experiences, who believed cancer is multi-causal, identified smoking as an addiction, or considered extrinsic factors responsible for smoking were more sympathetic.
3.	Wearn and Shepherd [80] UK/online	General population/quantitative randomized controlled trial (RCT)	Assessed impact of different framings of mass media news articles on stigma and cervical cancer screening intentions./Adapted from a previous study	They found that screening intention was positively associated with public stigma, self-stigma, perceived stigma, shame, and inferiority. Stigma toward people who had not been screened was greater when participants received an emotive narrative within a mass media news article (rather than factual information or no information) which in turn positively predicted the willingness to attend a cervical screening appointment. This suggests that one process through which emotive narratives within news articles promote screening is through increases in public stigma.
4.	Bresnahan *et al* [6] USA	General population/quantitative descriptive	Assessed differences in smoking and nonsmoking respondents’ attitude to lung cancer patients./Researchers developed	Nonsmoking respondents tended to stigmatise people with lung cancer, especially smokers who developed lung cancer.
5.	Shepherd and Gerend [62] USA	General population/quantitative RCT	Assessed attitude to cervical and ovarian cancer./Adapted from different scales	Findings from both studies suggested that people who were informed of the cause of cervical cancer felt more morally disgusted and 'grossed out', and were more likely to perceive a woman with cervical cancer as dirty, dishonest (men only) and unwise than people who were not informed of its cause.
6.	Myrick [51] USA/online	General population/quantitative descriptive	Assessed public perceptions and stigma due to listing cancer as the cause of death in celebrity obituary./Adapted from different scales	Their result suggests that lung cancer as a cause of death (as compared with liver cancer or death by an undisclosed cause) increased both anxiety and sadness, with anxiety resulting to increased origin-related stigma.
7.	Ongtengco *et al* [56] Senegal	General population/quantitative descriptive	Assessed cervical cancer stigma in non-patient population./Adapted from CASS	They found significant gender differences regarding cancer stigma. Women were significantly more likely than men to feel uncomfortable around someone with cancer, to hold the perception that once a person has cancer they can never be normal again, to feel that the needs of people with cancer should not be prioritised, to perceive that a cancer diagnosis was the fault of the individual and that cancer was more frightening than other diseases.
8.	Vrinten *et al* [77] UK	General population/quantitative descriptive	Quantified the prevalence and socio-demographic patterning of cancer stigma in the general population and to explore its association with cancer screening attendance./CASS	Higher stigma scores were associated with being male and being from an ethnic minority background. Higher total cancer stigma was sig associated with less likelihood of screening for cervical, breast and colorectal cancers./Low stigma.
9.	Azlan *et al* [3] UK/online	General population/quantitative RCT	Explore the role of disgust, in stigma towards people with cancer./CASS	Participants exposed to the cancer surgery video were more likely to experience greater disgust. Those experiencing greater disgust were also more likely to report greater avoidance- and awkwardness-based cancer stigma.
10.	Oystacher *et al* [58] South Africa	General population/	Examined the consequences of being labelled with a cancer diagnosis as barriers to accessing cancer treatment. NA	The study revealed three main labelling mechanisms: physical appearance of perceived signs/symptoms of cancer, which led to anticipated discrimination in response to prevalent cancer stereotypes and contributed to delayed treatment, use of traditional healers instead of biomedical treatment and secrecy of symptoms and/or diagnosis.
11.	Machado *et al* [42] Brazil	General population/qualitative	Examined the opinion of journalists, scientists and teachers about cancer./NA	The authors identified a negative view from professionals that may be contributing to or mirroring the vision of society that associates cancer with death and suffering. Words such as ‘cure’ are viewed with prejudice. On the other hand, a morbid approach arouses interest on the subject. It was also noted that the disclosure of a celebrity with cancer stands out as a decoy in the consumption of news. Such distortions may support actions that enhance communication about cancer, structured on pillars such as prevention, early diagnosis and cure.
12.	Zhang *et al* [91] China	Oral cancer/quantitative descriptive	Assessed the effects of stigma, hope and social support on quality of life (QoL) amongst Chinese oral cancer patients./SIS	Stigma was negatively related to QoL, explaining 39.3% of the variance./Low stigma
13.	Threader and McCormack [69] Australia	Head and neck cancer/qualitative	Explored the lived experience of head and neck cancer patients./NA	Despite the traumatic distress and stigma experienced by head and neck cancer patients, they developed positive changes as over time, previously unfelt empathetic understanding and altruism for others with cancer emerged from the impact of stigma on ‘self’. Also, acceptance triggered an awakening of new life interpretations and psychological growth in them.
14.	Yang *et al* [88] China	Prostate cancer/quantitative descriptive (prospective)	Examined if patients’ stigma, self-efficacy and anxiety mediate the relationship between doctors’ empathy and cellular immunity in patients with advanced prostate cancer treated by orchiectomy./SIS	The changes in patients’ stigma were statistically significant at admission, 14 days and 3 months. It was highest at 14th day and lowest at 3 months. Stigma had significant negative correlation with doctors’ empathy, patients’ self-efficacy and natural killer (NK) subset but positively correlated with anxiety.
15.	Wood [86] USA	Prostate cancer (& partners)/quantitative descriptive	Examined the relationships between stigma, QoL and relationships satisfaction for CaP survivors and their intimate/romantic partners./SIS	Stigma had significant negative correlation with QoL and relationship satisfaction./Low stigma.
16.	Wood *et al* [87] USA	Prostate cancer/quantitative descriptive	Investigated the influence of stigma on CaP survivors’ QoL./SIS	Stigma had signiﬁcant moderate influence on QoL and signiﬁcant negative correlation with each QoL subscale except the family/social subscale./Low stigma.
17.	Tripathi *et al* [71] India	Breast cancer/quantitative descriptive	Investigated the associations of high levels of stigma in women with breast cancer./BIABCQ	On multivariate logistic regression, with stigma as the dependent variable, being less educated and opting for BCS were associated with higher stigma./High stigma in 27.6%.
18.	Tsai *et al* [74] USA	Breast cancer/quantitative descriptive	Assessed the association between mainstream acculturation and QoL by investigating self-stigma, ambivalence over emotion expression (AEE) and intrusive thoughts./SSS	Mainstream acculturation was associated with lower self-stigma, which in turn was associated with lower AEE and intrusive thoughts, and subsequently resulted in lower QoL amongst Chinese-American BCS.
19.	Tsai and Lu [73] USA	Breast cancer/quantitative descriptive	Examined the relations between self-stigma and depressive symptoms, and further tested the influence of AEE and intrusive thoughts on self-stigma amongst Chinese-American BCS./SSS	Self-stigma was negatively correlated with annual household income and higher amongst those on chemotherapy than those without chemotherapy. Self-stigma was significantly associated with depressive symptoms amongst study participants with high levels of AEE and intrusive thoughts but not for those with low levels of AEE and intrusive thoughts.
20.	Wong *et al* [85] USA	Breast cancer/quantitative descriptive	Examined the association between self-stigma and QoL and tested the potential mediating roles of intrusive thoughts and posttraumatic growth in this relationship./Four items of the Chinese version of the SSS	Self-stigma was found to be negatively associated with QoL, and this association was mediated by more intrusive thoughts and less posttraumatic growth in a sample of Chinese-American BCS./Moderate stigma.
21.	Yeung *et al* [89] USA	Breast cancer/quantitative descriptive	Assessed the association between self-stigma and QoL and the mediating role of self-perceived burden./Four items of the Chinese version of the SSS	Self-stigma was significantly associated with higher self-perceived burden, poorer physical and emotional QoL as well as time since diagnosis amongst Chinese-American BCS.
22.	Nakash *et al.* [52] Israel	Breast cancer/quantitative descriptive	Examined the association between cancer stigma and QoL and the mediating role of pain intensity/CSI	Stigma amongst breast cancer patients was associated with worse QoL. Pain intensity partially mediated the relationship between cancer stigma and QoL.
23.	Trusson and Pilnick [72] UK	Breast cancer/qualitative	Explored women’s perceptions of social interaction during and after their treatment for early stage breast cancer./NA	Patients described the burden of the push towards positive thinking and the need to move on and get back to normal after treatment despite the continued association of cancer with death and the resulting potential for a stigmatised identity. They described accounts of significant others abandoning them at the time they needed them most. Other women described how they prioritised other people’s needs for comfort and reassurance over their own by playing down their private suffering and presenting a positive (public) image.
24.	Meacham *et al* [46] Uganda	Breast cancer/Qualitative	Examined the illness narratives of BCS. / NA	Stigma not only delayed women from engaging in care but also discouraged them from remaining in care through to treatment completion as the stigma could affect their marriage and family. Also, the cost of treatment paired with poor prognosis led to a stigma of draining family resources. The women coped through social support, maintaining positive outlook, acceptance of diagnosis accompanied by religious faith and ignoring negative comments that could erode their conﬁdence to continue treatment.
25.	Solikhah *et al* [92] Indonesia	Breast cancer/qualitative	Analysed the stigmatisation of breast cancer patients in Indonesia./NA	Indonesian women had negative perceptions towards breast cancer screening because of their experience of fear and shame. This made them to receive a complementary alternative treatment known as ‘kerokan’ and to consume white turmeric and Japanese ants. They coped through prayer and social support from family and other cancer survivors.
26.	Midding *et al* [48] Germany	Breast cancer/mixed methods	To investigate how male breast cancer patients feel about suffering from a ‘woman’s disease’./researchers developed	The highest stigma rate was found within the dimension having the feeling of being the only rooster in the yard beside all the women in breast cancer therapy (occurs in 18 men; 66.67%). Closely followed by the experience of sexual stigmatisation in the process of cancer care (16 men; 59.26%)./High stigma.
27.	Walker and Berry [79] USA	Breast cancer/qualitative	Explored the experiences of men with breast cancer (MBC)./NA	Three primary categories of experience were reported by MBC: (a) Feeling unwelcome in breast health centres; (b) Use of the term ‘chest cancer’ and (c) Becoming aware of other MBC. They recounted no visible signs in breast imaging centres indicating men belonged there as patients./High stigma.
28.	Gregg [28] Brazil	Cervical cancer/qualitative	Assessed how women with cervical cancer in Recife, Brazil endure and perpetuate stigma./NA	Cervical cancer in Recife was metaphorically loaded and heavily stigmatised. Women would not risk having their neighbours ﬁnd out they had cancer, for fear of being considered ‘spoiled’ or ‘ruined’. And doctors would not use the word ‘cancer’, choosing instead to use euphemisms like ‘inﬂammation’ or ‘wound’ as the word cancer is mystiﬁed as synonymous with death. The researcher observed that, rather than resisting stigma, the women with cervical cancer fortiﬁed stigmatising metaphors and blamed themselves, quite unjustly, for their own misfortune. The women thus accepted and coped with their lot in life by drawing on stigmatising metaphors to construct narratives that would help them understand the relationship between their new, ill, and, therefore, different selves and the world they have always known./High stigma.
29.	Dyer [18] USA	Cervical cancer/qualitative	This exploratory study examined the experiences of women who were survivors of cervical cancer, with a focus on possible stigmatisation relating to the release of the HPV vaccine and the increasing publicity surrounding cervical cancer’s connection to an sexually transmitted infection./NA	Participants felt that cancer as a whole was stigmatised through its enduring association with death and cervical cancer via its link with an STI. The media in promoting this view served as a ‘double-edged sword’ – increasing prevention behaviour whilst inadvertently increasing stigma against women with cervical cancer. Many assumed that others blamed them for having the disease. They felt so ashamed that they tell people they had uterine cancer instead of cervical cancer to avoid being judged. Patients cited the structural level manifestations of cervical cancer-related stigma and gave account of positive outcomes of cervical cancer-related stigma – chief of which is their own involvement in advocacy./High stigma.
30.	Maggio [43] USA/online	Lung cancer/quantitative descriptive	Determine the relationship amongst personal characteristics and lung cancer stigma, and the effects of stigma on psychosocial distress (i.e. anxiety and depression)./LuCaSS	Lung cancer patients with greater social constraints and lower self-esteem and who were smokers scored higher on stigma controlling for socio-economic status. Social support was a mediator for the relationship between stigma and depression but not for anxiety./Low stigma.
31.	Cataldo and Brodsky [11] USA/online	Lung cancer/quantitative descriptive	Investigated the relationship between LCS, anxiety, depression and physical symptom severity./Cataldo Lung Cancer Stigma Scale (CLCSS)	There were strong positive relationships between LCS and anxiety, depression and total lung cancer symptom severity. LCS provided a unique and significant 1.3% explanation of the variance in symptom severity beyond that of age, anxiety and depression./High stigma.
32.	Ostroff *et al* [57] USA/research electronic data capture	Lung cancer/quantitative descriptive	Examined group differences in lung cancer stigma for patients who report clinically significant depressive symptoms and established a suggested scoring benchmark to identify patients with clinically meaningful levels of lung cancer stigma./LCSI	Depressive symptoms were significantly positively correlated with lung cancer stigma and each of internalised stigma, perceived stigma and constrained disclosure irrespective of the smoking status. They found a statistically significant difference in lung cancer stigma between ever smokers and never smokers./High stigma.
33.	Liu *et al* [40] China	Lung cancer/quantitative descriptive	Examined the level of stigma and identify the correlates of stigma amongst lung cancer patients in China./SIS	Stigma was significantly and negatively associated with state self-esteem and coping self-efficacy./Moderate stigma.
34.	Johnson *et al* [37] USA	Lung cancer/quantitative descriptive	Identified lung cancer patients with high and low levels of stigma and examined the influence of stigma on social support, social constraints, symptom severity, symptom interference and QoL./Six-item stigma scale	Stigma was significantly related to lower levels of QOL. Those with high stigma had significantly higher symptom severity on feeling distressed, problems remembering things, and feeling sad, and greater symptom interference related to mood, relations with others and enjoyment of life. Participants also had significantly higher levels of social support and lower social constraints./High stigma in 35.5%.
35.	Rose *et al* [60] Australia	Lung cancer/quantitative descriptive	Explored help‐seeking behaviours, group identification, and perceived legitimacy of discrimination, and its potential relationship with perceived lung cancer stigma./CLCSS	Most sort help from the general practitioner (91.0%) and oncologist/treating clinician (81.3%) and more frequently used services providing assistance from health professionals (69.5%) and informational support (68.5%) than emotion-based support. Higher perceived lung cancer stigma was signiﬁcantly associated with greater perceived legitimacy of discrimination but not group identiﬁcation or help‐seeking behaviours./Stigma level was not indicated.
36.	Williamson et al [84] USA	Lung cancer/quantitative descriptive (prospective)	Tested if internalised lung cancer stigma and/or constrained disclosure were associated significantly with emotional and physical/functional QoL across 12 weeks in a sample of lung cancer patients on active oncologic treatment./Adapted from different scales	Internalised stigma and constrained disclosure were correlated significantly and did not interact significantly to predict emotional and physical/functional QoL. Higher internalised stigma and constrained disclosure were uniquely associated with poorer emotional and physical/functional well-being at study entry. Those who ever smoked (versus never smokers) reported higher levels of internalised stigma./High stigma.
37.	Steffen *et al* [65] Mexico	Lung cancer/quantitative descriptive	Examined how daily hope, defined as goal-directed effort and planning to meet goals, and daily stigma were related to same and next-day functioning in lung cancer patients receiving cancer treatment./Five items from CLCSS	At the between-person level, patients with higher levels of stigma did not report lower daily functioning. Within-person increases in stigma were related to lower social and role functioning regardless of physical symptoms. The effect of within-person increases in stigma was maintained in models that adjusted for negative affect; however, this effect did not carry into the next day once the previous day’s social and role functioning was included in the model.
38.	Maguire *et al* [93] UK	Lung cancer/quantitative descriptive	Investigated the prevalence of patient‐perceived lung cancer stigma and its relationships to symptom burden/severity, depression and deficits in health‐related QoL (HR‐QoL)./CLCSS	LCS was significantly correlated with younger age, greater social deprivation, being unemployed, depression, symptom burden and HR‐QoL deficits. Symptom burden explained 18% of variance in LCS. LCS explained 8.5% and 14.3% of the variance in depression and HR‐QoL, respectively./Low stigma.
39.	Carter-Harris [8] USA	Lung cancer/mixed methods	Examined the relationship of perceived lung cancer stigma and timing of medical help-seeking behaviour in symptomatic individuals. /CLCSS	The study reported a statistically significant positive correlation between perceived lung cancer stigma and delayed medical help seeking. In addition, smoking status was not related to perceived lung cancer stigma./High stigma.
40.	Occhipinti *et al* [55] Australia	Lung cancer (& caregivers)/qualitative	Examined the experiences of lung cancer patients and their caregivers and how stigma is manifested throughout a patient’s social network./NA	Patients and caregivers reported feeling high levels of felt stigma and concomitant psychological distress in response to the diagnosis of lung cancer. The study reported three overarching themes related to the nexus of lung cancer and smoking, the moralisation of lung cancer and smoking, and attacking the links between lung cancer and smoking. Furthermore, patients and caregivers commented on how smoking related imagery and lung cancer represented in public health advertisements tended to accentuate stigma. Both patients and their caregivers were ambivalent to stigmatising anti-smoking advertisements linked to lung cancer as some regard them as welcomed whilst others consider them as harsh and unnecessarily distressing./High stigma.
41.	Webb *et al* [81] USA, China, UK, Canada Australia and Korea	Lung cancer/scoping literature review (2000–2017)	Explored stigma in lung cancer patients with emphasis on how lung cancer stigma is measured, describe stigma experience of lung cancer survivors and effect of lung cancer stigma on survivors overall QoL./NA	The findings suggest that lung cancer stigma is a combination of perceived and internalised stigma stemming from the link between cigarette smoking and the disease itself. Also, individuals with lung cancer experience self-blame and guilt as well as altered QOL outcomes and depression, regardless of their history with tobacco use. Good healthcare provider communication was associated with decreased lung cancer stigma. When survivors perceive blame, responsibility or fatalism, positive communication is hindered. This may lead to delay in seeking medical assistance and concealment of symptoms that need assessment and management.
42.	Esser et al [23] Germany	Mixed cancer patients/quantitative descriptive	Investigated the effect of perceived stigmatisation on depressive symptomatology, body image and physical QoL across different cancer populations (Breast, prostate, colorectal and lung)./SIS	Stigmatisation showed total effects on depressive symptomatology across all stigma dimensions for all the cancer types except for lung cancer patients. Body image as a whole was shown to mediate the effect across all samples.
43.	Yilmaz *et al* [90] Turkey	Mixed cancer patients/quantitative descriptive	Determined the depression levels of adult oncology patients in the cancer treatment phase and identify both cancer-related stigma and the factors affecting their depression levels./questionnaire for measuring attitude towards cancer	A positive relationship was found between depression and attitudes toward cancer and its three domains. Almost half of the patients thought that they were discriminated against by employers and/or co-workers. Four factors indicating negative attitudes toward cancer were ‘being more than 60-year-old’, ‘higher education’, ‘low income’, and ‘feelings of social exclusion’, which accounted for 11% of the total./High stigma.
44.	Gökler-Danışman *et al* [26] Turkey	Mixed cancer patients/quantitative descriptive	Investigated the experience of grief by patients with cancer in relation to perceptions of illness, with a focus on the mediating roles of identity centrality, stigma-induced discrimination and hopefulness./Discrimination and stigma scale	They found that an increase in negative perceptions of the illness was associated with an increase in negative discrimination (enacted stigma), which in turn led to an increase in grief symptomatology. Thus, negative discrimination mediated in the relationship between illness perceptions and grief symptomatology.
45.	Ernst *et al* [22] German	Mixed cancer patients/quantitative descriptive	Investigated stigmatisation and its impact on QoL amongst a large sample breast, colon, lung and prostate cancer patients./SIS-D	They reported an inverse relationship between perceived cancer-related stigmatisation and various dimensions of QoL, with variation between cancer sites. Stigmatisation was lowest amongst prostate cancer patients. Stigmatisation predicted all five areas of QoL amongst breast cancer patients, but only affected emotional functioning amongst lung cancer patients./Moderate stigma.
46.	Shiri *et al* [64] Iran	Mixed cancer patients/quantitative descriptive	Determined stigma and related factors in individuals with cancer in Iran./Questionnaire for measuring attitude towards cancer	Of the participants, 57.5% agreed that their job performance would be reduced even after treatment, 54.5% considered it difficult to regain health after being diagnosed. There was a significant correlation between the stigma score and the level of education./High stigma in 26.1%.
47.	Moffatt and Noble [49] UK	Mixed cancer patients/qualitative	Explored the connections between cancer and employment and the constraints imposed by ill health and wider structural conditions./NA	Returning to work, for those who were able, helped repair the disruption caused by the illness. For those unable to work, reliance on welfare benefits, whilst necessary, conferred a stigmatised identity that compounded the disruption wrought by cancer. The felt stigma patients experienced was resisted by narratives of hard work and lifetime contributions to social security.
48.	Tang *et al* [67] Taiwan	Mixed cancer patients/qualitative	Explored the experience of stigma amongst female cancer patients./NA	The stigma of cancer includes the concepts of ‘cancer equals death’, ‘Cancer equals menace to social life’, ‘Cancer equals cancer-ridden life’, as well as being sensitive to the topics of death and calculating the number of remaining survival days.
49.	Stergiou-Kita et al [66] USA, Canada, Asia and Europe	Mixed cancer patients/scoping literature review (1980–2014)	Explored stigma and workplace discrimination as they relate to employment in working-age cancer survivors./NA	Myths regarding cancer such as its being contagious and will result in imminent death and that cancer survivors will be economic burden persist and can create misperceptions regarding survivors’ employability and lead to self-stigmatisation. Workplace discrimination may include hiring discrimination, harassment, job reassignment, job loss and limited career advancement. Strategies to mitigate stigma and workplace discrimination include education, advocacy and anti-discrimination policies.
50.	Harding *et al* [34] India	Stakeholders/qualitative	Developed an explanatory evidence-based model of stigma, communication and access to cancer palliative care in India which can be used to develop, test and implement future interventions./NA	The model explains how stigma associated with communicating a diagnosis of advanced cancer is enacted by treating oncologists, family members and community. This leads to patient expectations of cure and expensive futile treatment uptake that put them deeper into debt.
51.	Shiri *et al* [63] Iran	Stakeholders/qualitative	Examined the meaning of stigma and its effect on patients with cancer from the point of view of Iranian stakeholders./NA	Cancer was construed as a terrible and pitiful disease that cause communication breakdown, disease concealment and identity crisis.
52.	Nyblade *et al* [54] India	Stakeholders/qualitative	Examined the role of breast and cervical cancer related stigma from the perspectives of patients, community members and healthcare providers./NA	Participants in both studies voiced that cancer stigma is present in their lives and communities and is a barrier to screening, early diagnosis and treatment seeking for women with symptoms. Underlying reasons for cancer stigma emerging from the data revolved around: fear of contagion, the belief that cancer is transmissible; belief in personal responsibility for cancer; and cancer as incurable and the inevitability of an untimely death from it.
53.	Gupta *et al* [29] India	Stakeholders/qualitative	Evaluate cancer awareness and stigma from multiple stakeholder perspectives in North India, including men and women from the general population, health care professionals and educators, and cancer survivors./NA	The study found that most participants were unaware of what cancers are in general, their causes and ways of prevention. Attitudes of families towards cancer patients were observed to be positive and caring. Nevertheless, stigma and its impact emerged as a cross cutting theme across all groups. Cost of treatment, lack of awareness and beliefs in alternate medicines were identified as some of the major barriers to seeking care.
54	Mohabbat-bahar *et al* [50] Iran	Stakeholders/qualitative	Investigated stigma phenomenon, the process of formation and its impact on cancer patients and their families from the perspective of cancer patients, family members and oncology staff./NA	Results showed gradual process of cancer stigma formation and its different dimensions. Acceptance slightly leads to maintenance of adverse effects of stigma. Many patients admitted to having negative stereotypical beliefs before their cancer diagnosis and experienced stigma in form of negative reactions to themselves. Most patients cope via non-disclosure of cancer and limiting contact with others.
